# Prevalence and Risk Factors for Initiation of Smoking in Greek High-School Students

**DOI:** 10.3390/ijerph6030971

**Published:** 2009-03-02

**Authors:** Lazaros T. Sichletidis, Diamantis A. Chloros, Anastasios I. Tsiotsios, Dionisios G. Spyratos

**Affiliations:** Laboratory for the Investigation of Environmental Diseases, Pulmonary Clinic, Aristotle University of Thessaloniki, Thessaloniki, Greece; E-Mails: dchloros@msn.com (D.A.C.); atsiotsios@yahoo.gr (A.I.T.); dspyrato@yahoo.gr (D.G.S.)

**Keywords:** Adolescence, epidemiology, Greece, high-school students, smoking

## Abstract

The smoking habits of 9,276 high-school students (15–18 years old) in six cities of Northern Greece were studied using a questionnaire in order to determine the prevalence and possible risk factors for initiation of smoking. We observed that 29.6% of high-school students (32.6% of boys and 26.7% of girls) were current smokers. A percentage of 43.3% had started smoking before the age of 14. Reactive behaviour towards parents’ and teachers’ advice (40.2%) and the existence of smoking friends (40.1%) were the main reasons of initiation. A well-planned integrated anti-smoking campaign is urgently required, especially among students and teachers.

## 1. Introduction

Most data on the prevalence and the trends of smoking habits have been provided by the USA and only over the last years relevant research has been initiated in other countries, mostly within the framework of the worldwide anti-smoking campaign being carried out under the auspices of the WHO.

In Greece, the Ministry of Health and Welfare has recently implemented an anti-smoking campaign. The need of undertaking this new campaign has been identified by the identification of alarming new data, one of which is the first position of Greece among European Union countries in lung neoplasms in young men (<45 years old) with an annual incidence of 4.1 per 100,000 subjects of the population [1], whereas the mean incidence in the European Union is 2.5 per 100,000. The above increased rates keep pace with the increased annual per capita consumption of cigarettes by adults observed in Greece, which ranked 16^th^ worldwide in 1970–72 with 2,640 cigarettes and second with 3,590 cigarettes in 1990–92, according to WHO data [2].

The aim of this study was to determine the prevalence and risk factors for initiation of smoking among high-school students in six cities of Northern Greece. There are no recent epidemiological data on smoking habits concerning this age group in Greece and thus there is need for further epidemiological research. We hypothesized that high school students’ smoking status could predict future trends. Thus the implementation of proper preventative interventions appears to be obligatory.

## 2. Experimental Section

This study took place during a school year, from October 2001 to May 2002, among 9,276 high-school students (4,525 boys and 4,751 girls), aged 17.5 ± 1.3 years from six cities of Northern Greece (Thessaloniki 3,097 students; Kozani 2,441 students; Ptolemaida 1,356 students; Florina 1,011 students; Kastoria 771 students and Grevena 600 students).

In Northern Greece, there are several distinctive features concerning geographical and socioeconomic parameters. Population is extensively widespread and students move to bigger towns to study. There are 18 cities with a population of over 15,000 inhabitants. The six cities selectively chosen represent this socio-economic diversity. The most populated of them are Thessaloniki and Kozani. Thessaloniki is purely urban and the second biggest city in Greece, whereas Kozani is a regional urban city. Florina and Grevena have one of the lowest annual incomes per capita in Greece. Florina bases its economy on agriculture, while the basic occupation in the latter (Grevena) is stockbreeding. Ptolemaida is an important industrial area, where 75% of electricity of the country is produced. Finally, Kastoria used to be the main fur-producing city worldwide, with one of the greatest annual incomes per person. Using this selection together with the sample size, an acceptable representation of the Northern Greece population could be ensured.

Apart from Thessaloniki, questionnaires were administered in every high school of the remaining cities (Kozani, Florina, Ptolemaida, Grevena, Kastoria). In Thessaloniki, students from 15 out of 31 high schools (every other one) were included in the study. There is a numeric list (every school has a specific number) for all schools at the city of Thessaloniki and we decided to choose all schools with an even number. Every smoker filled in a questionnaire prepared by the Laboratory for the Investigation of Environmental Diseases, Aristotle University of Thessaloniki (Appendix). The study protocol has been reviewed and approved by the Scientific Committee of “G. Papanicolaou” Hospital, Thessaloniki as well as the Prefecture of Central Macedonia, Educational Department. The questionnaires were anonymous, however, parental informed consent was sought and obtained for all students. In addition, as the questionnaires were short and accurate in their content, nearly all students filled them in immediately. A total of 9,276 fully filled-in questionnaires were collected from a total of 9,456 initially handed out (98% response rate, mainly due to students’ absence from school on the day of collection). The questionnaire included questions concerning the initiation age, the number of cigarettes per day, the reasons for starting to smoke, the smoking habits of their parents, advertising and finally, the smoking hazards. As it has been proven with the use of biological indicators in the past, the adolescents usually answer honestly to such questionnaires [3].

According to the WHO glossary, “smoker” is someone who at the time of the survey smokes any tobacco product either daily or occasionally [4]. In the present study we defined the term of “smoker” as someone who smokes at least one cigarette daily. All others are regarded as non-smokers and handed in unanswered blank questionnaire, according to our instructions.

Comparison of frequency and severity of smoking between the two sexes was carried out using the *x^2^* test. Comparison of smoking initiation age was carried out using the *t* test.

## 3. Results and Discussion

Based on the analysis of the questionnaires, out of 9,276 high-school students who participated in the study 2,746 (29.6%) have been reported to be smokers. Out of 4,525 boys 1,477 (32.6%) have been stated to be smokers and out of 4,751 girls, 1,269 of them (26.7%) have been declared to be smokers, p<0,001. Table 1 shows the smoking habits of the students in every city. The average initiation age for boys was 14.4 ± 1.9 years and for girls 14.9 ± 1.6, p<0.001, *t*-test. We detected that 43.3% of the students reported that they started smoking before the age of 14 years.

Based on data concerning occupational status of inhabitants of these counties according to the last census of Greek population [5], we divided them into low, median and high financial position. In Thessaloniki, Kozani, Ptolemaida and Kastoria as a whole, we calculated that 41.5% of the population was in the lowest range, 45.8% in the middle and 12.7% in the highest one. On the other hand, in Grevena and Florina the percentages were 56.9%, 34.6% and 8.5% respectively. Comparing the four cities with the higher socioeconomic status with the other two, we found that students’ smoking was much more prevalent among the former (31.8% against 19.2%, chi-squared test p<0.001).

We observed that the majority of the students smoked 6 to 10 cigarettes per day (25.4%), whereas 20.7% smoked 16 to 20 cigarettes per day. Table 2 shows the number of cigarettes smoked per day. Despite the fact that the percentage of girls who smoke up to 20 cigarettes daily is higher compared to that of the boys, the percentage of boys who smoke more than 20 cigarettes is statistically significantly increased compared with girls (p<0.001).

A percentage of 36.7 (41.2% of boys and 30.9% of girls) reported that their parents are aware of the fact that they smoke. Taking all participants as a whole, the negative attitude towards parents’ and teachers’ advice (“reaction”) ranks first (40.2%), and the existence of friends who were in the habit of smoking was the second more common (40.1%, Table 3). Boys stated the existence of a friend who smokes as the main reason to start smoking (42% of cases) while the girls believe that they started to smoke as a reactionary behavior against parents and teachers (49.8% of cases). Concerning smoking habits of their relations, 28.6% stated that their father smoked, 20.1% their mother and 11.2% their favourite teacher. Finally, a percentage of 85.8% believe that young people are not influenced by the tobacco industry’s advertisements. The majority (95.2%) stated that they were aware of the health risks of smoking.

In a study conducted during 1989–90 in 2,032 high-school students in Athens, Patra and Ioannina [6] similar percentages of smokers were reported (33.5% of boys and 26% of girls). In a longitudinal study conducted on a representative Greek sample of adolescents, it was found that the prevalence of smoking hasn’t particularly changed since 1984, when 22% of them were smokers, until 1998 when 20.8% of those who answered the questionnaire were smokers [7]. It seems that in the last twenty years, smoking habits among Greek adolescents have remained quite steady.

In Budapest, according to a longitudinal study in students 14–18 year old, it was found that in 1995, 36% of them were smokers and 46% in 1999, i.e. an upward trend was observed [8]. In Slovakia the prevalence of smoking in adolescents was 24.8% in boys and 14.3% in girls, according to research conducted in 1999 on 1,571 subjects [9]. In the United Kingdom, 21% of the boys and 25% of the girls are smokers at the age of fifteen [10].

In the USA, it was detected that 20% of high-school students were current smokers in 2007 [11]. This percentage has remained quite stable since 2003, while there was no significant sex difference (21.3% of boys and 18.7% of girls in 2007). The percentage of students with current frequent cigarette use (>20 cigarettes per day) in the USA has been quite stable since 2003 (8.1% in 2007). In our study this percentage was estimated to be 5.7% of the students questioned. We should notice that the above study refers to high school students with no reference to exact age ranges.

The Global Youth Tobacco Surveillance (2000–2007), a survey that was conducted in 140 countries and 11 territories, concluded that 9.5% of students 13–15 year old smoke (12.1% of boys and 6.8% of girls) [12]. This percentage increases to 19.2% for the European region (21% of boys and 17.4% of girls) with the addition of 29.8% of never smokers who indicated that they were susceptible to initiate smoking during the next year. Nevertheless we should observe that this survey didn’t include data from western European countries.

It has been observed that the sooner someone starts smoking, the higher the cigarette consumption will be later in his life [13]. In our study it was found that the majority of the students started smoking before the age of 14, a very worrying finding. In accordance with our study, the average initiation age of smoking in Spanish primary education students was 11.6 years [14]. On the other hand, they smoke at a percentage of 12.1%. Based on the findings of the above studies, it is important for anti-smoking campaigns to be designed mainly targeted on young adolescents.

The higher prevalence of smoking in boys compared to girls is a consistent finding, not only of the specific epidemiological sample, but also observed in the majority of relevant studies [12]. However, over the last years the smoking prevalence in girls in some developed countries has reached or exceeded the relative smoking prevalence of boys [12,15,16], a change mainly attributed to the women’s liberation movement, through which smoking is expressed as an equity symbol being an exclusively men’s habit until recently [4]. In a recent study among female adolescents in Pakistan it was found that the prevalence of ever-smokers was 16.3%, a quite elevated percentage for a traditionally conservative society [17].

Smokers of both sexes answered that they started smoking mainly by reaction (“reaction” means a negative attitude towards parents’ and teachers’ advice; a kind of “revolution” against their ideas, totally expected behaviour concerning teenagers’ psychology) or because their friends used to smoke. Smoking as an attitude belongs to the framework of the physical development procedure of people and is closely related to the fundamental psychological characteristics of adolescence such as: search for new experiences, effort for independence from the parents, access to the group of people of the same age and the need to be accepted by them, search for identity, confirmation of their autonomy and revolutionary spirit [18,19]. At the same time friends, siblings and parents play a significant role in the behavior trends concerning smoking [20,21]. In the Global Youth Tobacco Surveillance study it was found that students in the European region were exposed to smoke in their home or public places in percentages of 77.8% and 86.1% respectively [13]. Moreover, adult prototypes like a teacher [21] or specific lifestyle aspects promoted by the mass media could create smoking trends [22].

A contributing factor to the spreading of smoking is the high standard of living. In our study, the prevalence is lower in the poorest cities of Florina and Grevena. This phenomenon has been described in Spanish adolescents where affluence is proportional to the habit of smoking [23].

Despite that 85.8% of smokers answered that they are not affected by the advertising launched by the tobacco industries, we should not overlook the general effect of advertising on young people. In a recent Cochrane Database review, that included nine longitudinal studies, it was concluded that exposure to tobacco advertising and promotion is associated with the likelihood that adolescents will start to smoke [24].

The majority of smokers answered that they were aware of the hazards of smoking. It seems that awareness of smoking hazards is inadequate in order to prevent the habit of smoking [12]. A recent meta-analysis of 23 randomized controlled trials showed that school-based programmes using a variety of interventions have only a borderline effect in preventing uptake of smoking [25]. New methods such as the peer-led intervention (i.e. training influential students to act as peer supporters during informal interactions outside the classroom to encourage their peers not to smoke) showed in a randomized controlled trial that it may be an effective preventive practice in the future [26].

The size of the study sample, the representative socio-economic composition of the sample as well as the fact that the study has shown that the current anti-smoking policy is unsuccessful are considered to be the main advantages of our study. However, the main limitations were the inability to include schools from all geographical regions of the country (due to technical difficulties) and the restricted size of the questionnaire, which promoted the success of the study but at the same time did not illustrate completely either the smoking habits or the detailed psychosocial causes of initiation of smoking.

## 4. Conclusions

It is concluded that the prevalence of smoking among high school students in Greece was higher than that of other developed countries. We believe that a well-planned integrated anti-smoking campaign, especially among students and teachers, is immediately required instead of a superficial awareness of the smoking hazards.

## Figures and Tables

**Table 1.** Smoking prevalence among students in six cities in Northern Greece.

	Thessaloniki n=3,097	Kozani n=2,441	Ptolemaida n=1,356	Kastoria n=771	Florina n=1,011	Grevena n=600	Total n=9,276
Smokers	sms %	sms %	sms %	sms %	sms %	sms %	sms %
Boys	477 33.4	408 35.5	243 32.7	172 42.9	137 27.1	40 17.2	1,477 32.6
Girls	525 31.5	330 27.0	159 25.9	123 33.2	80 15.8	52 14.1	1,269 26.7
Total	1,002 32.3	738 30.2	402 29.6	295 38.3	217 21.5	92 15.3	2,746 29.6

**Table 2.** Number of cigarettes smoked per day according to sex.

Number of cigarettes smoked / day	Boys (n=1,477) n %		Girls (n=1,269) n %		Total % (n=2,746)
1–5	186	12.6	249	19.6	15.8
6–10	325	22.0	372	29.3	25.4
11–15	266	18.0	254	20.0	18.9
16–20	294	19.9	275	21.7	20.7
21–25	133	9.0	49	3.9	6.6
26–30	93	6.3	28	2.2	4.4
31–35	28	1.9	12	0.9	1.5
36–40	86	5.8	15	1.2	3.7
41 +	66	4.5	15	1.2	3.0

**Table 3.** Reported reasons for smoking initiation.

Reason	Boys (%)	Girls (%)	Total (%)
“Reaction”	33.6	49.8	40.2
Their friends smoke	42.0	38.3	40.1
Because their “models” smoke	26.5	22.6	24.9
Because they like the idea of a young trendy person smoking	18.9	19.0	18.9
Because they think it’s “cool”	14.8	6.5	11.5
Because they want to convince themselves and the others that they have grown up	6.2	2.1	4.5

**“Reaction”** means a negative attitude towards parents’ and teachers’ advice.

**“Models”** are the adult models like the teacher or other model types promoted by the media and particularly by television who create trends and are perceived as identification models from the adolescents.
